# FKBP8, a new member of the PIK3C3/VPS34 complex

**DOI:** 10.1080/27694127.2022.2100041

**Published:** 2022-07-21

**Authors:** Milton Osmar Aguilera, María Isabel Colombo

**Affiliations:** a Consejo Nacional de Investigaciones Científicas y Tecnológicas (CONICET). Buenos Aires, Argentina; b Laboratorio de Mecanismos Moleculares Implicados en el Tráfico Vesicular y la Autofagia. Instituto de Histología y Embriología de Mendoza (IHEM), Universidad Nacional de Cuyo-CONICET. Mendoza, Argentina; c Facultad de Odontología, Universidad Nacional de Cuyo, Microbiología, Parasitología e Inmunología, Mendoza, Argentina

## Abstract

Macroautophagy/autophagy is an adaptable pathway involved in the degradation of very different targets that include proteins, organelles, or even invading intracellular microorganisms. The regulation of this complex pathway depends on a great number of proteins, some common for the majority of the processes and others specific for a particular autophagic event. Nevertheless, the kind of interaction between the players contributes to determining the specificity of the regulation. In a recent study, we found a new regulatory protein of starvation-activated autophagy called FKBP8. The absence of this protein impairs autophagy activation produced by serum starvation and its overexpression can activate the pathway in cells incubated in full media. Besides, we found that the FKBP8 function is mediated by the interaction with the PIK3C3/VPS34-containing complex. Previously, FKBP8 has been shown to participate in mitophagy. In the latter process, FKBP8 works inducing mitochondrial fission, and also it functions as a receptor protein through its LIR domain to direct autophagy. In contrast to mitophagy, in starvation-activated autophagy, not the LIR but the transmembrane domain of FKBP8 is necessary for the regulatory function and interaction with the PIK3C3 complex.

## Article

Macroautophagy (hereafter autophagy) is a vesicular transport pathway whose main function is the degradation and recycling of intracellular components. The first observations of this pathway were made through electron microscopy where material with similar characteristics to the cytoplasm was found inside vesicles. This observation suggested a mechanism of sequestering and degradation of intracellular components. Subsequently, some decades later, biochemical assays were performed on liver tissue confirming this degradation pathway. But it was not until the late 1990s that the first specific marker for autophagosomes, called Apg8/Aut7 in yeast, and later the mammalian homolog MAP1LC3/LC3, was found. Several autophagy genes were subsequently identified in yeast as well as their orthologs in more complex eukaryotic cells. This discovery produced an exponential increase in the study of this pathway. Autophagy has been observed to be involved in a wide variety of processes in a list that continues growing to this day. In this way, it has been determined that the activation of the autophagic pathway responds to a large number of stimuli such as protein starvation, the absence of glucose, hypoxic conditions, oxidative stress, organelle damage or malfunctioning, and even the presence of microorganisms in the cell. Likewise, the targets of degradation are diverse and specific to each stimulus and cell lineage, ranging from soluble proteins to almost the entire cell, as in the case of maturation from erythroblast to erythrocyte. To react to this multiplicity of processes, cells use a complex regulatory network where many components are ubiquitous and others are specific for each particular process. One example is the degradation of ubiquitinated targets, where different receptor proteins recognize ubiquitin modification, but all of these receptor molecules have an LC3-interacting region (LIR) that binds LC3. In this way, a wide range of different substrates are targeted to a phagophore.

In our article [1]⁠ we describe a new regulator protein involved in starvation-mediated autophagy. FKBP8 is critical during the starvation-mediated induction of autophagy. Typical induction of the autophagy pathway by serum depletion is avoided in both transiently depleted FKBP8 cells using an siRNA and in stable *FKBP8* knockout cells generated by the CRISPR-CAS9 system. We arrived at this conclusion by monitoring via western blot analysis, the MAP1LC3B lipidation in the presence or absence of the lysosomal inhibitor chloroquine, and by quantifying the MAP1LC3B-positive dots number by fluorescence microscopy. In contrast, an increase in the protein level by overexpression results in autophagy activation similar to a starvation stimulus.

Recently, it has been described that FKBP8 has a role in mitophagy. When we analyzed in our system the extension and functionality of the mitochondrial network, no differences are observed in cells overexpressing FKBP8 in comparison with untransfected cells. This finding suggests that the effect observed on autophagy when we overexpress FKBP8 is not mainly due to mitophagy activation in our system.

Then, we performed a detailed colocalization analysis using markers of different stages in the autophagic pathway. We initially performed colocalization studies between MAP1LC3B and FKBP8 (endogenous and overexpressed protein) to identify the autophagy stage at which this immunophilin might participate. Interestingly, only partial and relatively low colocalization levels of MAP1LC3B and FKBP8 are observed. As a rule, the latter phenomenon occurred at the edge of RFP-MAP1LC3B-positive vesicles. Likewise, low levels of colocalization are observed for FKBP8 and RAB7A, which is a protein that is present at the late autophagosome/autolysosome, suggesting that FKBP8 does not play a role in the late stages of autophagy.

Previous work has demonstrated that FKBP8 is present at the endoplasmic reticulum (ER) and mitochondria. The ER is one of the sites where autophagosome formation takes place. Based on these data, we carried out colocalization experiments of FKBP8 with proteins that are present in the ER, such as CALR (calreticulin) and SEC61A1. Interestingly, the starvation stimulus induces a statistically significant increase in the colocalization levels of FKBP8 with both proteins, suggesting that this protein plays a role in autophagy at this organelle.

Afterward, we investigated if FKBP8 plays a role at the level of the PIK3C3 complex, analyzing the colocalization between this protein and two members of the complex, ATG14 and BECN1. The PIK3C3 complex activation, and the subsequent generation of PtdIns3P, is one of the earliest steps in the autophagic pathway. Our findings indicate that these two proteins markedly colocalize with FKBP8 under both full nutrient and starvation conditions. In addition to colocalization analysis between BECN1 and FKBP8, we performed both co-immunoprecipitation and FRET experiments and demonstrated that FKBP8 and BECN1 are part of the same complex. Interestingly, we observe an increase in the kinase activity when FKBP8 is overexpressed. This increase is evinced by a higher ZFYVE1 dots number, a specific PtdIns3P-binding protein in the autophagic pathway. Otherwise, diminution in protein level using the transfection with an siRNA against *FKBP8* impairs the starvation-induced increase of ZFYVE1 dots.

Two proteins, ZFYVE1 and WIPI1 or WIPI2, that bind PtdIns3P, which is the product of the PIK3C3 complex activity, are recruited early to autophagosome primordial structures. Despite the low levels of colocalization, it is noteworthy that ZFYVE1 and WIPI1 are found in proximity to FKBP8-positive structures. Interestingly, time-lapse video microscopy studies reveal a transient colocalization of ZFYVE1 and FKBP8. Taken together, these results suggest that FKBP8 plays a role during the early steps of autophagosome biogenesis. However, this protein is excluded from the formed vesicles, implying that FKBP8 is involved in steps that are upstream of autophagosome generation

Finally, we explored the FKBP8 protein sequence looking for a domain that might be important in its regulatory function in starvation-induced autophagy. The LIR domain present in the amino-terminal portion of the protein is necessary for FKBP8 function in mitophagy. However, deletion of the FKBP8 amino-terminal portion does not affect starvation-mediated autophagy nor the interaction with BECN1, suggesting that another domain is important for this specific function. This effect is in agreement with our results showing no modification in the mitochondrial network both, in extension and functionality, in cells overexpressing FKBP8.

FKBP8 is the only member of the FK506 family of proteins that possesses a transmembrane domain, which is in charge of the protein positioning in the cell. Interestingly, we found that the transmembrane domain deletion in FKBP8 affects the interaction with BECN1. In agreement with these results, the overexpression of this truncated protein diminished the activation of autophagy generated by the starvation stimulus.

**Figure 1.**
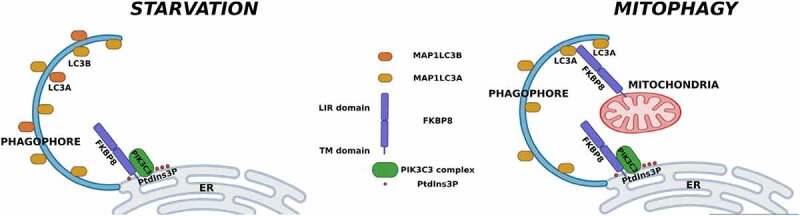
FKBP8 regulated autophagy in several process using different domains.

All these results point to FKBP8 as a scaffold protein that positions the PIK3C3 complex specifically in starvation-activated autophagy. More importantly, we can infer that FKBP8 may play several roles in the autophagic pathway using different domains, depending on the activation stimulus (Fig.1).
